# In silico prediction of optimal multifactorial intervention in chronic kidney disease

**DOI:** 10.1186/s12967-025-06977-3

**Published:** 2025-08-21

**Authors:** Agnieszka Latosinska, Ioanna K. Mina, Thi Minh Nghia Nguyen, Igor Golovko, Felix Keller, Gert Mayer, Peter Rossing, Jan A. Staessen, Christian Delles, Joachim Beige, Griet Glorieux, Andrew L. Clark, Joost P. Schanstra, Antonia Vlahou, Karlheinz Peter, Ivan Rychlík, Alberto Ortiz, Archie Campbell, Harald Rupprecht, Frederik Persson, Harald Mischak, Justyna Siwy

**Affiliations:** 1 Mosaiques Diagnostics GmbH, Rotenburger Straße 20, 30659 Hannover, Germany; 2 Institute for Molecular Cardiovascular Research, University Hospital RWTH Aachen, Aachen, Germany; 3 Department of Internal Medicine IV (Nephrology and Hypertension), Medical University Innsbruck, Innsbruck, Austria; 4 Steno Diabetes Center Copenhagen, Herlev, Denmark; 5 Department of Clinical Medicine, University of Copenhagen, Copenhagen, Denmark; 6 Non-Profit Research Institute Alliance for the Promotion of Preventive Medicine, Mechlin, Belgium; 7 School of Cardiovascular and Metabolic Health, University of Glasgow, Glasgow, UK; 8 Division of Nephrology and KfH Renal Unit, Hospital St Georg, Leipzig, Germany; 9 Martin-Luther University Halle, Wittenberg, Germany; 10 Department of Internal Medicine and Paediatrics, Nephrology Section, Ghent University Hospital, Ghent, Belgium; 11 Hull and East Yorkshire NHS Hospitals Trust, Castle Hill Hospital, Cottingham, UK; 12 Institute of Cardiovascular and Metabolic Disease, Institut National de La Santé Et de La Recherche Médicale (INSERM), U1297 Toulouse, France; 13 Université de Toulouse, Toulouse, France; 14 Centre of Systems Biology, Biomedical Research Foundation of the Academy of Athens, Athens, Greece; 15 Atherothrombosis and Vascular Biology Program, Baker Heart and Diabetes Institute, Melbourne, VIC Australia; 16 Department of Physiology, Anatomy, Microbiology, La Trobe University, Melbourne, VIC Australia; 17 Department of Medicine and Immunology, Monash University, Melbourne, VIC Australia; 18 Department of Cardiometabolic Health, University of Melbourne, VIC, Australia; 19 Department of Internal Medicine, Third Faculty of Medicine, Charles University, and University Hospital Královské Vinohrady, Prague, Czech Republic; 20 Instituto de Investigación Sanitaria de La Fundación Jiménez Díaz (IIS-FJD) UAM, Madrid, Spain; 21 Centre for Genomic and Experimental Medicine, Institute of Genetics and Cancer, University of Edinburgh, Edinburgh, UK; 22 Department of Nephrology, Angiology and Rheumatology, Klinikum Bayreuth GmbH, Bayreuth, Germany; 23 Department of Nephrology, Medizincampus Oberfranken, Friedrich-Alexander-University Erlangen-Nürnberg, Erlangen, Germany; 24 Kuratorium for Dialysis and Transplantation (KfH) Bayreuth, Bayreuth, Germany

**Keywords:** Chronic kidney disease, Clinical proteomics, Drug response prediction, Optimizing intervention, Urine peptides

## Abstract

**Background:**

Chronic kidney disease (CKD) contributes to global morbidity and mortality. Early, targeted intervention can help mitigate its impact. CK273 is a urinary peptide classifier previously validated in a prospective clinical trial for the early detection of nephropathy. We hypothesized that drug-induced molecular changes in the urinary peptidome could be predicted in silico and guide selecting interventions for individual patients.

**Methods:**

The efficacy of the urinary peptidomic classifier CKD273 in predicting major adverse kidney events (≥ 40% decline in estimated glomerular filtration rate or kidney failure -median follow-up: 1.50 (95%CI 0.35, 5.0) years), was confirmed in a retrospective cohort of 935 participants. In silico prediction of treatment effects from four drug-based interventions (Mineralocorticoid receptor antagonist, Sodium-glucose co-transporter 2 inhibitor, Glucagon-like peptide-1 receptor agonist, and Angiotensin receptor blocker), dietary intervention (olive oil), and exercise was performed based on: a) individual baseline urinary peptide profiles, and b) previously defined fold changes in peptide abundance after treatment in clinical trials. Following recalibration to align with outcomes of these trials, CKD273 scores were calculated for each patient after in silico treatment. For combination treatments, the effects of multiple interventions were combined.

**Results:**

Simulated interventions demonstrated a significant reduction in median CKD273 scores, from 0.57 (IQR: 0.19–0.81) before to 0.039 (IQR: −0.192–0.363) after the most beneficial intervention (paired Wilcoxon test, *P* < 0.0001). The combination of all available treatments was not the most frequently predicted optimal intervention. Patients with higher baseline CKD273 scores required more complex intervention combinations to achieve the greatest score reduction.

**Conclusions:**

This study supports the feasibility of in silico predicting effects of therapeutic interventions on CKD progression. By identifying the most beneficial treatment combinations for individual patients, this approach paves the way for precision medicine trials in CKD. A prospective study is currently being planned to validate the in silico-guided intervention approach and determine its exact benefits on patient-relevant outcomes.

**Supplementary Information:**

The online version contains supplementary material available at 10.1186/s12967-025-06977-3.

## Background

The development of multiple different treatment options for chronic kidney disease (CKD) has resulted in a major change of patient management. In the past, treatment was primarily focused on managing the underlying risk factors, such as hypertension and diabetes, through the use of antihypertensive and anti-diabetic therapies. Angiotensin-converting enzyme inhibitors (ACE inhibitors) and angiotensin receptor blockers (ARBs) were commonly prescribed to control blood pressure, one key driver of CKD progression [1]. These treatments, while beneficial in slowing disease progression in some cases, are not addressing the complex pathophysiology of kidney damage. Recently, multiple novel drug classes were introduced, all demonstrating benefit in reducing progression in CKD. Sodium-glucose co-transporter 2 inhibitors (SGLT2i), initially developed for the management of type 2 diabetes, reduce albuminuria, protect against decline in kidney function, and improve cardiovascular outcomes in patients with CKD, both with and without diabetes [2–4]. These effects are thought to be mediated by mechanisms such as improved glomerular hemodynamics, reduced inflammation, and enhanced tubuloglomerular feedback [5]. Another recent development is the use of the non-steroidal mineralocorticoid receptor antagonist (MRA), finerenone, [6]. which targets the mineralocorticoid receptor more selectively than traditional MRAs such as spironolactone. MRAs may help counteract the fibrosis and inflammation seen in CKD progression [7]. Glucagon-like peptide-1 (GLP-1) receptor agonists, such as semaglutide, reduce albuminuria and improve kidney outcomes, particularly in obese patients with diabetes [8]. Their effects are thought to be due to a combination of metabolic benefits, especially weight loss and improved insulin sensitivity. Endothelin-1 receptor antagonists are another class of drugs under investigation for CKD treatment. Endothelin-1 is a potent vasoconstrictor that contributes to kidney fibrosis and glomerular injury. Blocking the endothelin receptor, endothelin-1 antagonists, in combination with ARBs, may reduce kidney injury and progression in various forms of CKD [9]. Initial findings suggest that endothelin-1 antagonists could offer additional benefits for patients with specific CKD phenotypes. Additional evidence also supports non-drug interventions such as dietary and lifestyle changes in the management of CKD [10, 11].

These multiple treatment options highlight the need for specific, personalized treatment guidelines for CKD. Personalized treatment based on specific molecular features is by now routine in oncology, [12]. with treatment targeting specific molecular changes. In contrast, due in part to the absence of a specific mutation causing CKD (with some rare exceptions like e.g. autosomal dominant polycystic kidney disease or Alport syndrome), personalized interventions are generally not implemented in nephrology. While pharmaceutical companies, in their own interest, often promote a “one-size-fits-all” approach, advocating the use of their respective drug in every patient, this strategy leads to an increased risk of adverse drug interactions, higher healthcare costs (e.g. yearly costs of Sparsentan, for the treatment of primary immunoglobulin A nephropathy, exceed 50,000 €, [13]), and potential side effects, especially in a population already at high risk of comorbidities. Moreover, clinical evidence supporting the combined use of all these agents is generally moderate, inconclusive or non-existent. For example, studies examining the combination of GLP-1 receptor agonists with SGLT2i have failed to demonstrate an added benefit in kidney protection, [8]. raising questions about the optimal use of combination therapies in CKD management.

We have developed a urine peptide-based classifier, CKD273, which allows for early detection of CKD and provides prognostic information regarding disease progression [14, 15]. This classifier uses peptidomic analysis of urine to capture molecular signatures of kidney dysfunction and predict outcomes. In parallel, we have investigated how various therapeutic interventions affect the urine peptidome [16–20]. Using computational models, prediction of the effects of individual treatments was demonstrated as a feasible approach [21]. The data generated from these studies indicate substantial variability in treatment responses in individuals, underscoring the need for personalized treatment strategies. What works best for one patient may not be effective in another, even if their clinical phenotype is overall quite similar.

**Fig. 1:**
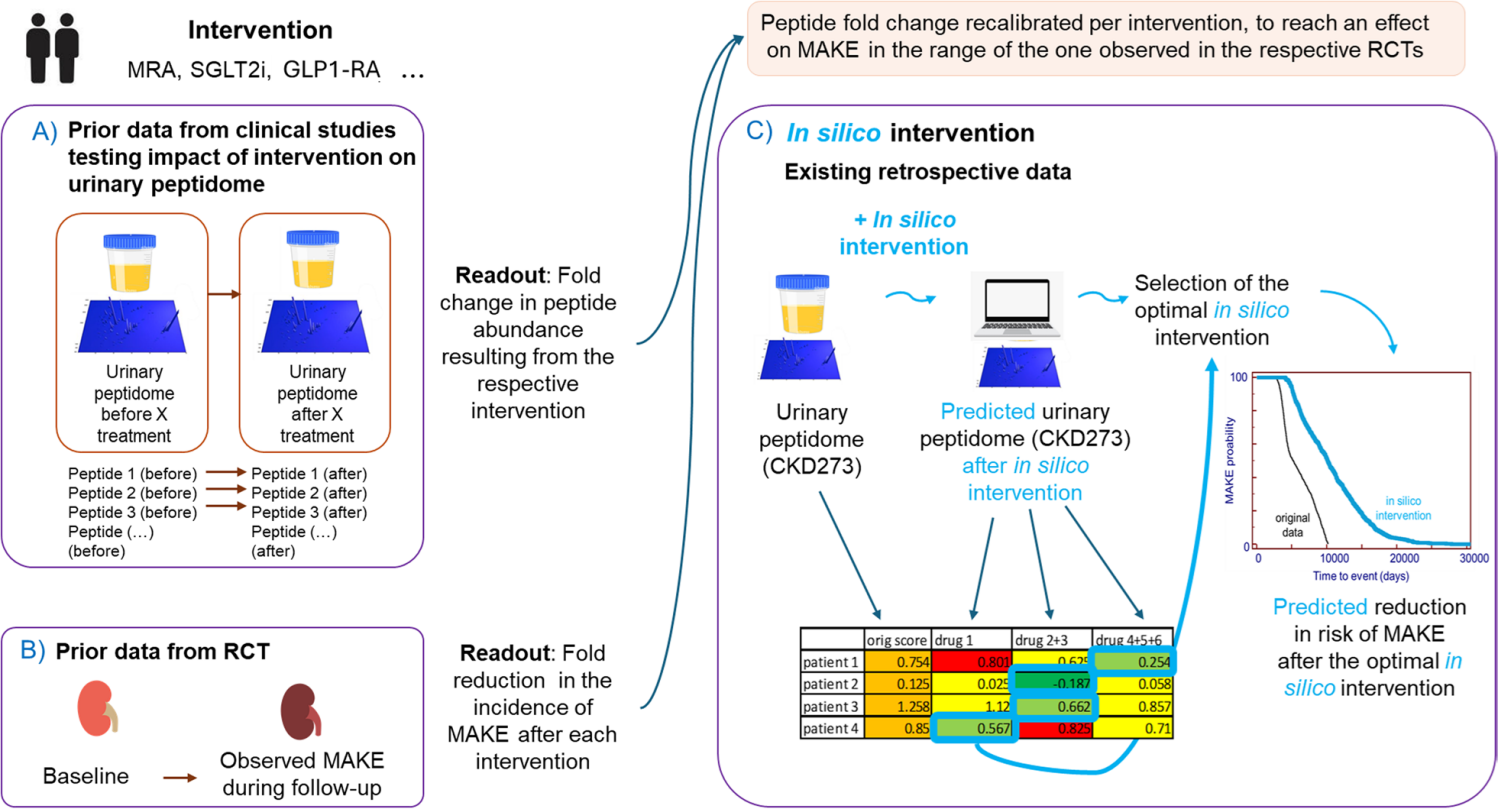
Overview of the approach. Different types of intervention were applied in silico on urinary peptidomics data to predict the impact of treatment, with the extent of the impact being calibrated to fit the observed effect in large randomized trials. The personalized optimal combination of interventions was determined in 935 patients, based on the impact on CKD273. The results indicate that guiding personalized treatment based on urine peptide data is possible and may substantially improve patient management

Here we describe a predictive framework based on CKD273 scoring that enables clinicians to design personalized treatment regimens that maximize therapeutic efficacy while minimizing the risk of adverse effects (Fig. 1). We hypothesized that molecular changes in the urinary peptidome can predict the most beneficial combination of therapeutic interventions for individual patients. To test this hypothesis, we investigated the in silico molecular effects of six different therapeutic interventions, in all possible combinations, on the urine peptidome to identify the optimal treatment for each patient.

## Methods

### Study participants and study design

This study includes datasets from 935 patients from previous studies on diabetes and CKD: PRIORITY, DIRECT, PREDICTIONS, SUNmacro, and CACTI. All datasets have been generated using the same analytical platform, capillary electrophoresis coupled to mass spectrometry, and processed using a consistent data processing pipeline to ensure comparability [22, 23]. Specifically, following data acquisition, peptide mass, migration time, and signal intensity were calibrated using internal urinary standard peptides [24]., thus minimizing batch-related peptide variation [22, 25]. Previous studies have shown that the utilised pre-processing approach does not affect the predictive performance of the CKD273 model [26, 27]. Detailed information on the designs and the methods used in these studies are available in previous publications [15, 28–31]. Inclusion criteria were availability of relevant demographic and clinical variables including age, sex, body mass index, blood pressure, estimated glomerular filtration rate (eGFR, calculated using the CKD Epidemiology Collaboration, CKD-EPI, 2009 formula), increased risk of CKD based on CKD273 scoring > 0 at the time of the baseline assessment, and availability of follow-up information (eGFR or kidney failure at follow-up). The endpoint was major adverse kidney event (MAKE), defined as a decline of ≥ 40% in eGFR values during follow-up or kidney failure. The study was conducted according to the guidelines of the Declaration of Helsinki. All datasets were fully anonymized and from previous studies. The ethics committee of the Hannover Medical School Germany waived ethical approval under the reference number 3116–2016 for all studies involving re-use of data from anonymized urine samples.

### Peptide-based classifiers and prediction of events

The previously developed classifier CKD273 was used for prediction of CKD events and assessment of the impact of different interventions and their combinations. The scores for the classifier were predicted by a support vector machine (SVM) algorithm, integrated into the MosaDiag software, [32] in which the levels of 273 CKD biomarkers detected in the urine of individual patients are compared to their respective levels in CKD. Sample classification was conducted at baseline and after in silico intervention. All statistical tests were performed in MedCalc version 12—© 1993–2013 MedCalc Software.

### In-silico impact of intervention

**Fig. 2:**
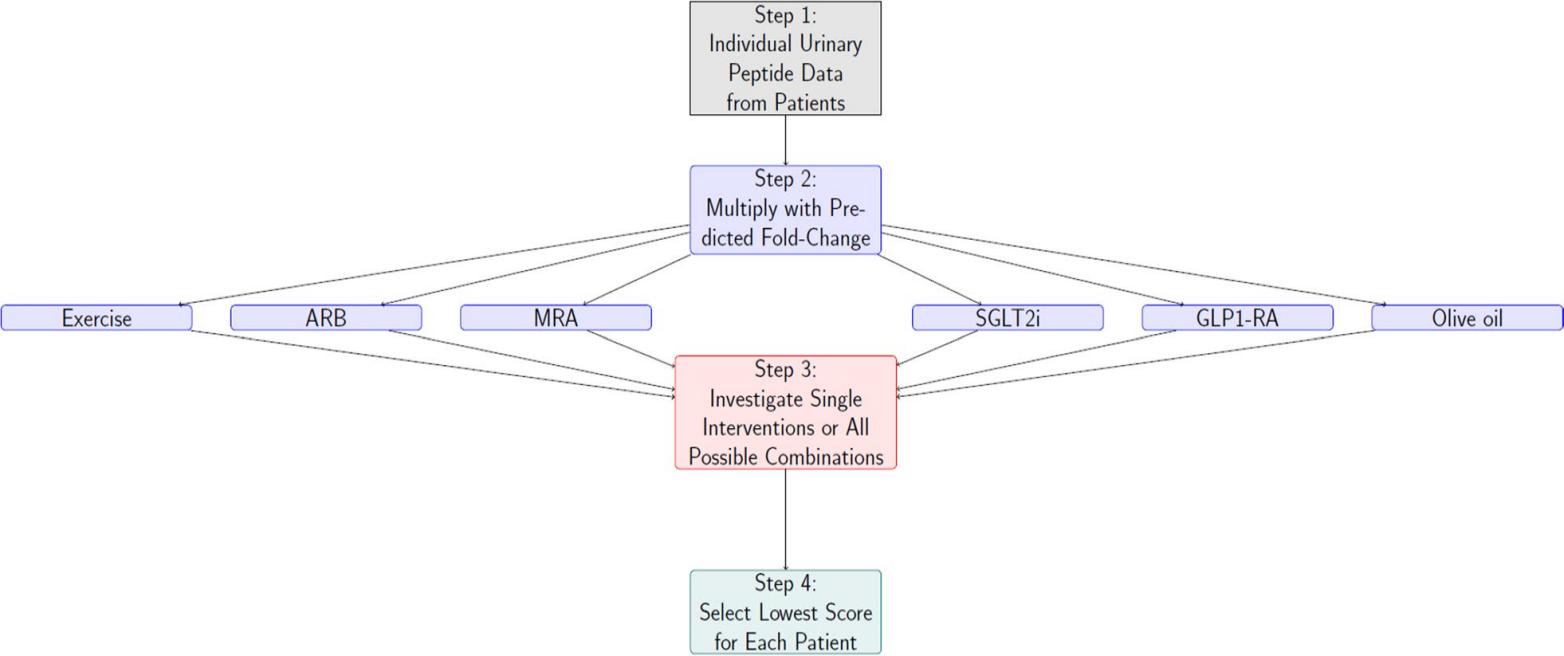
Graphical depiction of the study outline. First, urinary peptide data are acquired from patients (Step 1), which then undergo in silico simulated treatment using single (Step 2) or multiple (Step 3) interventions to predict the impact of interventions on individual urinary peptides. In silico predictions are based on past experience with patients that had urinary peptide assessments prior to and after initiation of treatments in clinical studies. In the last step the optimal intervention, resulting in the maximal in silico lowering of the risk (lowest urinary CKD273 peptidomic classifier score), is chosen

We assessed the impact on urinary peptidomic profiles of four different drug-based interventions (MRAs, SGLT2i, GLP1 receptor agonists, and ARBs); one dietary intervention (olive oil); and one lifestyle intervention, exercise. The data on the treatment induced impact were generated in previous studies either published [16–20, 33]. or unpublished (exercise). Treatment effect on peptide abundance was quantified by the fold changes, given in Supplementary Table S1. Fold changes were calculated by dividing the average peptide abundance after treatment by the average peptide abundance before treatment (representing a result of the intervention). Missing abundance values, in general the result of the signal below detection limit, were replaced by zero. Since the follow-up after treatment was in the range of 6 weeks to 9 months, but in general not until MAKE occurred and to limit overestimation of the treatment effect, the applied fold-changes were recalibrated per treatment, ensuring that the effect on MAKE was within the range observed in the respective randomized controlled trials [2, 6, 8, 34]. Specifically, for each treatment, we calculated the ratio between the observed effect in the trial (percentage reduction in time to MAKE) and the predicted effect from our model (percentage reduction in time to 50% MAKE risk). This ratio was then used as a correction factor and applied to adjust the fold changes for each peptide. To simulate the effect of intervention, these re-calibrated fold changes were then applied to multiply the intensities of the respective peptides in each patient, and the predictor (CKD273) scores were re-calculated. When applying a combination of different interventions, similar principle was followed, with the effects combined (via multiplication of all relevant fold changes) (Fig. 2).

### Transformation of the CKD273 biomarker score into risk of MAKE

To make CKD risk prediction more tangible and link it to a measure of risk of MAKE, the CKD273 score was transformed into the incidence rate of MAKE per 100 person-years (based on the data available from [21]). For each CKD273 score, the incidence rate per 100 person-years was calculated as follows: 100 subjects with the closest CKD273 scores were selected for each target score, comprised of 50 individuals with scores above and 50 with scores below the target score value. Subsequently, for each subset of 100 subjects the sum of observation times was divided by the observed number of subjects experiencing a MAKE, resulting in the average predicted time until MAKE. Assuming linear development, by dividing this value by 2, the expected time to a 50% risk of MAKE in a cohort with a specific CKD273 score was derived (Supplementary Table S2).

The relationship between the CKD273 score and the estimated time to a 50% risk of MAKE was then assessed using regression analysis to establish the following association: Log “Days to 50% risk of MAKE” = 4.1446–0.9498*CKD273 score. The derived formula was subsequently used to transform both original CKD273 scores and scores modified after in silico interventions.

## Results

### Cohort characteristics

Based on the applied criteria, 935 datasets were retrieved, corresponding to 935 individuals. The subjects were predominantly male (73%), diabetic (97.8%) and obese (median body mass index (BMI): 30 kg/m^2^). The majority of participants presented with established impaired kidney function, as indicated by a median eGFR of 46 mL/min/1.73 m^2^. The age distribution ranged from 25 to 87 years, with a median age of 63 years. The median CKD273 score was 0.57 (95% confidence interval (CI): 0.02, 1.02). The median follow-up time was 1.50 years (interquartile range (IQR): 1.00–2.92). A total of 71 CKD events were recorded during follow-up. The occurrence of CKD events across the cohorts did not show statistically significant differences in incidence rates (*p* = 0.3), suggesting a comparable risk distribution. Furthermore, no significant differences were observed in other relevant clinical or demographic variables between the cohorts (Supplementary Table 3). The summary clinical and demographic variables are listed in Table 1, and in Supplementary Table S2 on an individual level.

**Table 1:** Descriptive statistics for the participants providing the urine samples (n = 935) analysed within the study

Duration of follow-up (years)	1.50 (0.35, 5.0)
Clinical characteristics	
Age (years)	63 (45, 78)
Female, n (%)	255 (27.3%)
SBP (mm Hg)	138 (109, 160)
DBP (mm Hg)	77 (56, 95)
Hypertension, n (%)	461 (49.4%)
Diabetes, n (%)	914 (97.8%)
eGFR (mL/min/1.73 m2)	45.95 (19.57, 113.38)
BMI (kg/m^2^)	30.3 (21.7, 44.8)
CKD273 score	0.565 (0.02, 1.02)

Categorical variables are described with absolute (N) and group-wise relative frequencies (%), continuous variables with median (95% CI). BMI body mass index, CI Confidence Interval, DBP diastolic blood pressure, eGFR estimated glomerular filtration rate, SBP systolic blood pressure

### Event prediction using CKD273 classifier

As a first step the relationship between the peptide-based classifier CKD273 and the risk of MAKE was investigated. The data were categorized into tertiles based on their CKD273 classifier scores, enabling stratification into low, intermediate, and high-risk subgroups. The incidence of MAKE progressively increased across the tertile subgroups. A distinct stepwise rise in risk was observed, with individuals in the highest tertile showing significantly higher event rates compared to those in the lowest tertile (P < 0.0001, hazard ratio 4.26 (95%CI: 2.35–7.73), Supplementary Figure S1). This observation confirms the predictive power of the CKD273 classifier, demonstrating its capacity to effectively differentiate between patients with varying degrees of risk for a kidney event.

### Reduction of CKD273 scoring following in silico intervention

The CKD273 score was assessed for patients at baseline and following in silico treatment. Each single intervention and all possible combinations were investigated. In total, this process resulted in the in silico application of 63 different therapeutic regimens. The comprehensive range of simulated treatments allowed for the tentative identification of the most effective intervention tailored to each individual patient's molecular profile.

**Fig. 3:**
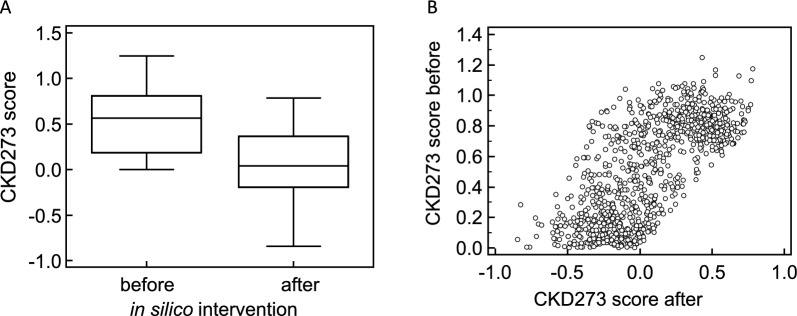
Assessment of CKD273 scores before and after in silico intervention. Box-and-whisker plots (**A**) and scatter plot (**B**) illustrating the distribution of CKD273 scores before and after the in silico intervention. The scatter plot represents CKD273 scores before (as measured in urine of patients) in the vertical axis and CKD273 scores after (as predicted by in silico interventions) in the horizontal axis. Note the different scale of the two axes. For in silico-treated subjects, the intervention that resulted in the lowest CKD273 score was plotted

For the in silico -treated subjects, the intervention yielding the lowest CKD273 score was selected as the optimal intervention for maximizing CKD risk reduction. CKD273 scores before and after the in silico interventions are detailed in Supplementary Table S4. Following the predicted most beneficial intervention, there was a reduction in the median CKD273 score from 0.57 (IQR: 0.19–0.81) before treatment to 0.039 (IQR: −0.192–0.363) after treatment (paired Wilcoxon test, *P* < 0.0001) (Fig. 3A). There was a clear shift in the distribution of scores toward negative values (Fig. 3B), indicating a potential reduction of CKD progression. For every subject, at least one intervention or combination of interventions successfully reduced the CKD273 score. The mean decrease in CKD273 score (delta) before and after treatment, based on the lowest score achieved, was 0.45. The analysis revealed that the impact of interventions was significantly associated with the baseline CKD273 score. Patients with higher baseline scores exhibited a greater absolute reduction in CKD273 scores following in silico interventions (rho = −0.355, p < 0.0001), likely reflecting a higher burden of active pathological processes at baseline.

**Fig. 4:**
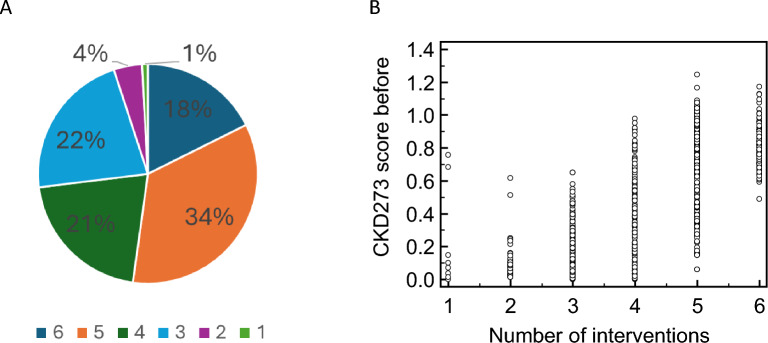
The number of interventions predicted to be applied in combination for the optimal treatment strategy. **A** Pie chart showing the frequency distribution of the predicted optimal number of intervention combinations per patient (*n* = 935). **B** Plot illustrating the association between the baseline CKD273 score of patients prior to the intervention and the number of interventions predicted to be applied in combination to result in the lowest CKD273 score post-intervention

The number of patients benefiting most (based on the largest reduction in CKD273) from a specific intervention (single or combination) is shown in Table 2. Interestingly, the combination of all available treatments was not the most frequently predicted as optimal intervention (Fig. 4A). Instead, specific combinations, such as MRA + SGLT2i + ARB, were frequently predicted as optimal interventions compared to single or double interventions. Patients for whom in silico interventions predicted treatment with a higher number of drugs tended to have higher CKD273 scores before treatment (Fig. 4B). This suggests that individuals with a higher initial risk required more complex combinations of interventions to achieve the maximal reduction in CKD273 scores.

**Table 2:** Overview of the number of subjects corresponding to each predicted optimal interventions, and the combinations of interventions that resulted in the maximal reduction of risk

Intervention	# of subjects
MRA + Exercise + GLP1RA + SGLT2I + ARB	320
MRA + Exercise + GLP1RA + SGLT2I + Olive oil + ARB	165
MRA + SGLT2I + ARB	123
MRA + Exercise + SGLT2I + ARB	120
MRA + Exercise + ARB	48
MRA + Exercise + GLP1RA + ARB	39
MRA + GLP1RA + SGLT2I + ARB	31
MRA + ARB	16
MRA + GLP1RA + ARB	16
SGLT2I + ARB	15
Exercise + GLP1RA + ARB	8
ARB	4
GLP1RA + ARB	4
GLP1RA + SGLT2I + ARB	4
MRA + GLP1RA + SGLT2I	4
Exercise + GLP1RA + SGLT2I + ARB	2
GLP1RA + SGLT2I	2
Olive oil	2
MRA + Exercise + SGLT2I + Olive oil + ARB	2
MRA + SGLT2I	2
Exercise + GLP1RA + SGLT2I + Olive oil + ARB	1
Exercise + SGLT2I + ARB	1
GLP1RA	1
SGLT2I	1
MRA + Exercise + GLP1RA + SGLT2I	1
MRA + Exercise + SGLT2I	1
MRA + GLP1RA + Olive oil + ARB	1
MRA + SGLT2I + Olive oil + ARB	1

ARB angiotensin receptor blockers, GLP1RA Glucagon-like peptide-1 receptor agonist, MRA mineralocorticoid receptor antagonist, SGLT2I sodium-glucose co-transporter 2 inhibitor

### *Reduction in Risk of MAKE following *in silico* intervention*

**Fig. 5:**
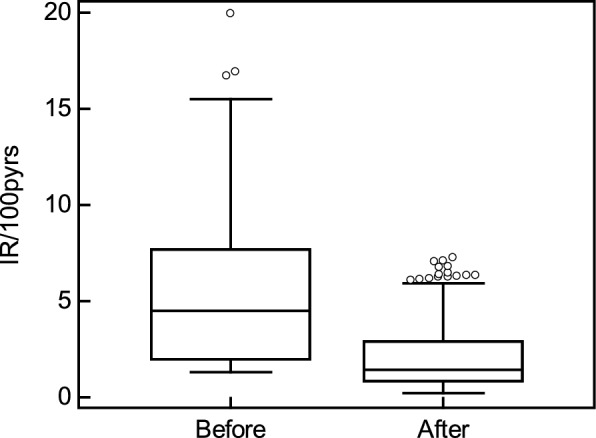
Box-and-whisker plots illustrate the incidence rate of major adverse kidney events (MAKE) per 100 person-years before and after the in silico intervention. A significant decrease is observed following the application of the in silico intervention (paired Wilcoxon test, *P* < 0.0001), indicating a reduction in risk after the intervention. Abbreviations: IR- incidence rate, pyers- person years

The relationship between the estimated time to a 50% risk of MAKE and the baseline CKD273 score for the subjects in the study is displayed in Supplementary Figure S2. The relation was used to develop a predictive model to estimate the time to a 50% risk of MAKE, followed by the incidence rate of MAKE per 100 person-years, based on the CKD273 score. The respective CKD273 scoring and calculations are given in Supplementary Table S4. Based on this transformation, the median incidence rate per 100 person-years risk of MAKE was 4.5 at the baseline and 1.43 after the predicted optimal intervention (Fig. 5), with a median relative risk reduction of 61% (Supplementary Table S4). Briefly, if a study followed 1,000 people for one year, we would expect about 45 new cases of MAKE without and 14 after the application of the in silico intervention, based on the median incidence rates.

## Discussion

Prediction of response to intervention has been identified as a key topic in patient management, with proteomics holding the promise of providing solutions [35]. As one first step in this direction, the PRE-score has been introduced by the group of Heerspink and colleagues to predict response to therapy, based on selected clinical variables [36]. The availability of a plethora of different drugs and drug classes has nurtured interest in the concept of multifactorial intervention in CKD in recent years, driven by evidence suggesting that addressing multiple pathways simultaneously can slow progression, reduce complications, and improve quality of life. The 2024 KIDGO guidelines [37]. list 4 pharmacological interventions to treat CKD and the list is likely to grow given ongoing clinical trials and additional kidney protective drugs already approved for specific indications, frequently at very high cost. However, the optimal composition and implementation of such interventions remain underexplored.

In this study we investigated an alternative approach for the management of CKD, based on molecular signatures. CKD remains a major public health challenge, predicted to become the 5th global cause of death by 2040 [38, 39]. This study addresses the urgent need for guidance on personalized interventions for CKD patients. While the benefit is not formally demonstrated in a prospective trial, it could serve as guidance for treatment, which is currently lacking. It additionally may guide the design of future clinical trials [15]. The goal is to move away from a “trial-and-error” approach towards a precision medicine model for CKD management, where treatments are not only based on clinical features but are also guided by molecular insights specific to each patient. This is expected to significantly improve patient outcomes by tailoring interventions to the individual's disease profile, optimizing the use of available therapies, and potentially delaying or even preventing the need for kidney replacement therapies such as dialysis. As the field of CKD treatment continues to evolve, personalized strategies based on molecular profiling, such as those derived from the urine peptidome, hold promise to become a cornerstone of clinical practice.

The theoretical background, to adjust treatment based on the individual molecular phenotype, is already in place in oncology [40]. Nephrology faces a similar dilemma: while multiple drugs are available, there is lack of sufficient guidance on which one to use and how to effectively combine them [37]. However a genetics-based treatment adjustment strategy as in oncology is not applicable to CKD. As such, it seems reasonable to start an approach in a similar direction: to obtain guidance on the optimal intervention from the patient’s molecular phenotype, however, in this case based on urinary peptide signatures that reflect non-invasively the “status” and (patho)physiology of the kidney [41–43].

By developing an in silico treatment profile for each patient, the effects of intended treatments and their combinations can be efficiently simulated based on retrospective proteomic substance-related outcome data. Our approach was based on fold change multiplication to simulate the combined effects of various interventions. This pragmatic method was chosen due to the lack of peptidomics data illustrating the impact of multiple interventions on peptide levels, and for its scalability in simulating numerous interventions in silico.

Validated in a prospective trial and supported by the FDA for use in early kidney disease detection during drug development, CKD273 composite score captures the complexity of CKD by integrating multiple molecular markers, which represent different pathological mechanisms of the disease. Unlike single biomarkers, this approach provides a more comprehensive view of CKD. The classifier can distinguish between low- and high-risk individuals and show its utility as a stratification tool for personalized intervention strategies [15]. The ability to simulate the effects of drugs on this composite score, reflecting their influence on multiple molecular pathways, allows for predictions of therapeutic impact, based on molecular changes. The proposed approach provides information on each patient for all combinations of interventions, enabling clinicians to make tailored decisions. Even though specific interventions could be excluded based on patient characteristics, risks, or contraindications, based on all available data the optimal treatment excluding the specific intervention can still be defined. It is important to acknowledge that the underlying peptide shifts used for simulation were derived from real-world interventional trials in which CKD273 was measured before and after treatment. Thus, the molecular changes applied to simulate therapeutic effects are empirically based. However, since these data were used to estimate the predictive framework, they cannot serve as an independent validation. A prospective trial will be required to confirm the predictive utility of this approach.

The study has several limitations, in part also owed to its novelty:

1. The in silico intervention is based on previously collected data, many from moderate size studies. Future investigations of larger cohorts with a longer follow-up time should enable prediction of the drug effect with higher accuracy.
2. Possible confounders that may impact the drug effects are not known and were therefore not taken into account, neither were albuminuria or background treatment at the time of sampling.
3. The approach assumes that the treatment effects are independent and additive, due to lack of data on potential interactions between interventions. All interventions were therefore considered independent from each other. This approach may, in some cases, affect the accuracy of the predicted outcomes, thus further validation is planned to address these limitations.
4. The study is observational, a causal relationship between peptidomic changes and clinical outcomes was not investigated and is not implied.
5. This is an in silico study, the exact benefit of the approach can only be assessed based on prospective trials, comparing clinical randomized trials with the in silico guided intervention.
6. The MRA tested in urinary peptidomics was spironolactone, a steroidal MRA while the approved drug to treat CKD is finerenone, a nonsteroidal MRA.
7. Due to the retrospective nature of the study and limited covariate data, adjustment for potential confounders such as comorbidities, medications, or lifestyle factors was not possible.
8. Important clinical, lifestyle, genetic, and epigenetic factors influencing CKD progression, such as blood pressure, weight, diet, smoking, and metabolic control, were not assessed due to limited data availability. Their impact should be evaluated in future prospective studies.

## Conclusions

In this study, we demonstrated the feasibility of a robust, pragmatic and effective approach using the individual, molecular peptidomic signature of a patient to guide treatment in CKD. The study was based on the hypothesis that a pharmaco-omics approach, in this case based on urine peptides, should enable guiding a personalized approach towards management of CKD. An apparent major benefit of the approach is that due to the high dimensionality (over 20,000 peptides in urine are described and assessed), the theoretical benefit of not just one, but of combined therapeutic interventions can be predicted. In addition, impact of additional treatments (e.g. novel drugs) can be added to the approach, enabling expansion with moderate efforts. Our data describe a potential profiling tool towards optimisation of CKD treatment. A prospective study is currently being planned to validate the in silico-guided intervention approach and determine its exact benefits on patient-relevant outcomes. This is even more important considering the availability of multiple drug classes for intervention, the lack of guidance selecting the most appropriate compounds and the high costs for novel drugs.

## Supplementary Information

Additional file1

Additional file2

## Data Availability

Anonymized data used for the analyses will be made available upon request directed to the corresponding author. Proposals will be reviewed and approved by the authors with based on scientific merit and feasibility. After approval of a proposal, data can be shared via a secure online platform after signing a data access and confidentiality agreement. Data will be made available for a maximum of 5 years after a data sharing agreement has been signed.

## Abbreviations

ACE: Angiotensin-converting enzyme
ARB: Angiotensin receptor blocker
BMI: Body mass index
CI: Confidence interval
CKD: Chronic kidney disease
eGFR: Estimated glomerular filtration rate
GLP-1 RA: Glucagon-like peptide-1 receptor agonist
IQR: Interquartile range
MAKE: Major adverse kidney event
MRA: Mineralocorticoid receptor antagonist
SGLT2i: Sodium-glucose co-transporter 2 inhibitor
SVM: Support vector machine

## Declarations

### Generative AI and AI-assisted technologies

During the preparation of this work the author(s) used [ChatGPT by OpenAI, Grammarly]. in order to improve readability and language, including grammar. After using this tool, the author(s) reviewed and edited the content as needed and take(s) full responsibility for the content of the publication.
