# Supplementary material for: In silico prediction of optimal multifactorial intervention in chronic kidney disease
Source: J Transl Med. 2025 Aug 21;23:943. doi: 10.1186/s12967-025-06977-3 (PMC12372250; doi:10.1186/s12967-025-06977-3)
Supplement: Supplementary file 1 — Additional file1 [file 12967_2025_6977_MOESM1_ESM.pdf]

Supplementary Figures

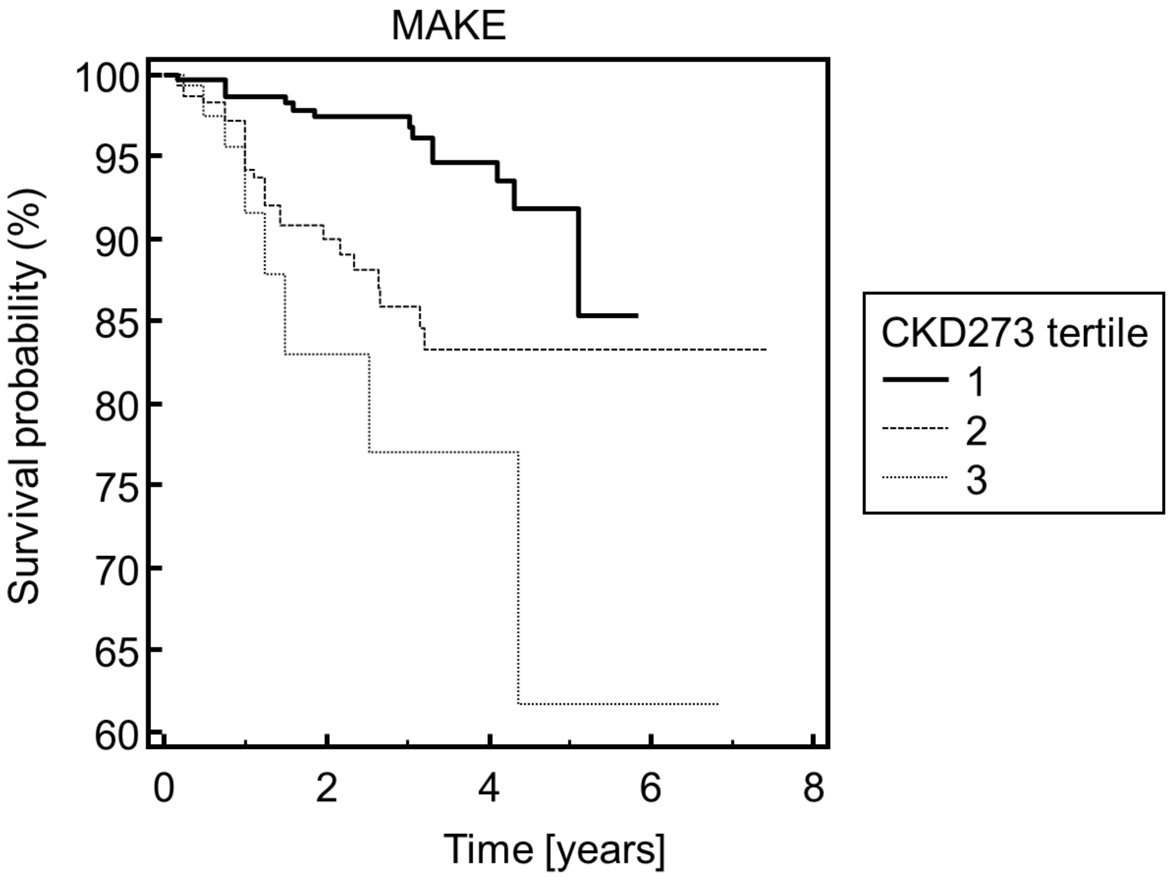

Supplementary Figure 1: *The relationship between the baseline CKD273 score and MAKE. Shown is the Kaplan–Meier plot for the CKD outcome (MAKE) with CKD273 classification scores from the lowest (1) to highest (3) tertile representing the low, intermediate, and high-risk subgroups (P < 0.0001. Abbreviations: MAKE – major adverse kidney event.*

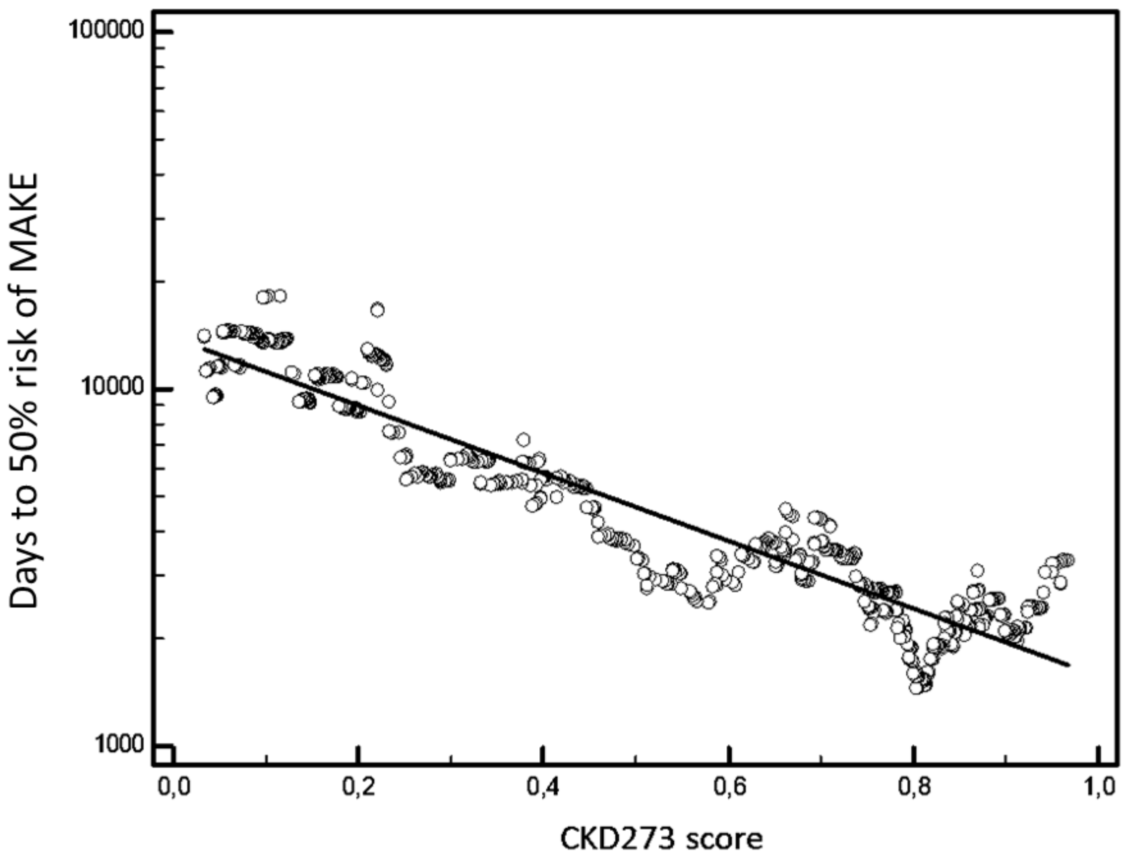

Supplementary Figure 2: *The relationship between the baseline CKD273 score and the estimated time to a 50% event rate of MAKE (p<0.001). Abbreviations: MAKE – major adverse kidney event.*
